# Künstliche Intelligenz und Ethik im Gesundheitswesen – Spagat oder Symbiose?

**DOI:** 10.1007/s00103-022-03653-5

**Published:** 2023-01-17

**Authors:** Dennis Küster, Tanja Schultz

**Affiliations:** Cognitive Systems Lab, Universität Bremen, Enrique-Schmidt-Str. 5, 28359 Bremen, Deutschland

**Keywords:** Worst-Case-Szenarien, Autonomie, Lebensspanne, Assistenzsysteme, Demenz, Worst-case scenarios, Autonomy, Lifespan, Assistance systems, Dementia

## Abstract

Künstliche Intelligenz (KI) gewinnt auch im Gesundheitswesen immer mehr an Bedeutung. Diese Entwicklung löst ernst zu nehmende Sorgen aus, die sich anhand von sechs großen „Worst-Case-Szenarien“ zusammenfassen lassen. Von einer KI-basierten Verbreitung von Desinformationen und Propaganda über einen möglichen militärischen Wettlauf zwischen den Großmächten bis hin zu einer möglichen Herrschaft der Algorithmen („Algokratie“) auf Basis einer voreingenommenen Torwächterintelligenz: Die realen Gefahren einer unkontrollierten weiteren Entwicklung von KI sind insbesondere im Gesundheitsbereich keinesfalls zu unterschätzen. Allerdings könnte der Menschheit aus Angst vor KI jedoch die Möglichkeit entgehen, die Entwicklung unserer Gesellschaft gemeinsam mit uns freundlich gesinnter KI positiv zu gestalten.

Anwendungsfälle im Gesundheitswesen spielen in diesem Diskussionsbeitrag eine vorrangige Rolle, da hier sowohl die Risiken als auch die Chancen neuer KI-basierter Systeme besonders deutlich werden. Dürfen z. B. ältere Menschen mit Demenz (MmD) Teile ihrer Autonomie KI-basierten Assistenzsystemen anvertrauen, damit sie andere Aspekte ihres Alltagslebens weiterhin selbstständig meistern können? In diesem Beitrag argumentieren wir, dass sich der Spagat zwischen Ethik und KI sowie den Gefahren und Chancen von KI im Gesundheitswesen zumindest teilweise durch einen langfristig angelegten ethischen Ansatz in Richtung einer Symbiose zwischen Mensch und KI überwinden lässt. Wir illustrieren diesen Ansatz beispielhaft anhand unseres „I-CARE“-Systems, eines KI-basierten Empfehlungssystems zur tertiären Prävention von Demenz. Dieses System wurde seit 2015 im gleichnamigen Projekt „I-CARE“ an der Universität Bremen entwickelt und wird dort bis heute erforscht..

## Abstract (en)

Artificial intelligence (AI) is becoming increasingly important in healthcare. This development triggers serious concerns that can be summarized by six major “worst-case scenarios”. From AI spreading disinformation and propaganda, to a potential new arms race between major powers, to a possible rule of algorithms (“algocracy”) based on biased gatekeeper intelligence, the real dangers of an uncontrolled development of AI are by no means to be underestimated, especially in the health sector. However, fear of AI could cause humanity to miss the opportunity to positively shape the development of our society together with an AI that is friendly to us.

Use cases in healthcare play a primary role in this discussion, as both the risks and the opportunities of new AI-based systems become particularly clear here. For example, would older people with dementia (PWD) be allowed to entrust aspects of their autonomy to AI-based assistance systems so that they may continue to independently manage other aspects of their daily lives? In this paper, we argue that the classic balancing act between the dangers and opportunities of AI in healthcare can be at least partially overcome by taking a long-term ethical approach toward a symbiotic relationship between humans and AI. We exemplify this approach by showcasing our I‑CARE system, an AI-based recommendation system for tertiary prevention of dementia. This system has been in development since 2015 as the I‑CARE Project at the University of Bremen, where it is still being researched today.

## Einleitung

In Hollywoods Filmstudios begegnen uns seit Jahrzehnten dystopische Zukunftsvisionen, in denen Künstliche Intelligenz (KI) übermenschliche Intelligenz erlangt oder Roboter als mysteriöse und gefährliche Wesen dargestellt werden [1]. Nicht selten versuchen diese gar, die Menschheit auszulöschen [2]. Während sich solche düsteren „Worst-Case-Szenarien“ in den Warnungen einiger KI-Ethiker widerspiegeln [3], argumentieren andere, dass die Invasion der Roboter in unserem Alltag längst Realität sei [4].

Tatsächlich sind gewisse KI-gestützte Algorithmen bereits weit verbreitet: Wir kommunizieren mit ihnen online und lassen uns von ihnen beraten. Wir nutzen KI wie selbstverständlich, um uns in unseren immer komplexer werdenden digitalisierten Lebensbedingungen weiterhin zurechtzufinden [4]. Folgt man Autoren wie David Gunkel [4], so wird sich hier kein plötzlicher Moment der Machtübernahme durch die Maschinen ereignen. Stattdessen findet eine langsame, aber stetige Invasion statt. Aus dieser Perspektive stellt sich die Frage, wie wir mit diesen Veränderungen langfristig und vorausschauend umgehen [4] und unter welchen Umständen Mensch und Maschine in Zukunft Verbündete sein können [5].

Beim Blick auf die Chancen und Gefahren der Zusammenarbeit zwischen Menschen und KI verdient die wachsende Bedeutung von KI im Gesundheitswesen ein besonderes Augenmerk. Diese wird beispielsweise in den Bereichen der Befundauswertung, Datenanalyse und Public Health immer häufiger eingesetzt. Doch welche ethischen Fragen stellen sich konkret in diesem Bereich?

In diesem Beitrag möchten wir insbesondere die Rolle kognitiver Systeme beleuchten, die in der Unterstützung besonders Gefährdeter einen enormen positiven Beitrag leisten könnten. Zu dieser Gruppe gehören Menschen mit Demenz (MmD). Die Anwendung kognitiver Systeme bei dieser Indikation schien bisher oft einen Spagat zwischen KI und Ethik darzustellen, dagegen könnte deren nachhaltige Allianz [5] oder „Symbiose“ letztlich helfen, menschliche Autonomie zu stärken, statt sie zu gefährden. Vor einem näheren Blick auf die Chancen einer solchen Allianz sollten in der ethischen Abwägung die potenziellen Gefahren von KI klar benannt werden. Zu diesem Zweck stellen wir zunächst die aus unserer Sicht prominentesten Worst-Case-Szenarien für die Folgen von KI dar und beziehen diese auf einen möglichen Einsatz im Gesundheitswesen. Auf dieser Grundlage entwickeln wir dann ein „Best-Case-Szenario“ für eine „symbiotische KI“, die menschliche Autonomie unterstützt. Wir illustrieren unsere Vision anhand des I‑CARE Projekts, das MmD mit Hilfe eines auf der Basis nachvollziehbarer ethischer Prinzipien entwickelten Systems unterstützen soll. Abschließend diskutieren wir vor diesem Hintergrund die Frage zur Rolle von KI im Gesundheitswesen: Um den Spagat zwischen KI und Ethik sowie Chancen und Gefahren aufzulösen, bedarf es ethisch geprüfter Ansätze, in denen KI Menschen im Erhalt ihrer Autonomie unterstützt.

## Worst-Case-Szenarien

Unabhängig vom Einsatzfeld umreißen KI-Experten sechs große Prototypen realer Worst-Case-Szenarien für KI [2], die sich auch beim Einsatz von KI im Gesundheitswesen widerspiegeln könnten:

### 1. Massive Verbreitung von Desinformation.

KI-gestützte Systeme sind in der Lage, Desinformationen in einem zuvor ungeahnten Maßstab zu generieren [6]. Wo zuvor seit Jahrtausenden solche absichtlich in die Irre führenden Falschinformationen allein durch Menschen, ob einzeln oder in Gruppen, verbreitet wurden, kann KI nun Lügen und Desinformationen in einem globalen Maßstab produzieren. Sprachsysteme wie GPT‑3 (Generative Pre-trained Transformer 3) können maschinelle Lernverfahren wie „Deep Learning“ ähnliche Texte wie Menschen produzieren [7]. Sie sind bisher vermutlich noch nicht leistungsfähig genug, um selbstständig Desinformationskampagnen zu führen [6]. Jedoch können sie den durch menschliche Betreiber verursachten Schaden erheblich vergrößern, indem sie eine Flut von Desinformationen generieren.

Dies betrifft auch und insbesondere den Gesundheitsbereich. Diese Form von KI könnte z. B. dazu missbraucht werden, massenhaft Desinformationen zu Themen wie den vermeintlichen Gefahren von Impfungen oder einem angeblichen Missbrauch persönlicher Gesundheitsdaten durch den Staat zu verbreiten. So könnten Desinformationen sehr schnell zu einem enormen Schaden für die öffentliche Gesundheit führen. Demnach dürfte es in kaum einem sonstigen Bereich so gefährlich werden wie im Gesundheitsbereich, wenn wir es zulassen, dass Fiktion unsere Realität bestimmt.

### 2. Bedrohung der Bevölkerungen und Gesellschaften.

Wenn KI für nationale Sicherheitsinteressen relevant wird, könnte es leicht zu einem gefährlichen Geschwindigkeitswettbewerb beim Erreichen bestimmter automatisierter Systemfähigkeiten kommen [8, 9]. Dies könnte katastrophale Folgen haben, bis hin zu kriegerischen Auseinandersetzungen, falls die sicherheitsgerechte Entwicklung z. B. von militärischen KI-Systemen dabei untergraben wird [2].

Im Gesundheitsbereich entstehen global betrachtet keine derart dramatischen Worst-Case-Szenarien. Dennoch gibt es auch hier Einwände, die gegen eine Abgabe von Autonomie an Roboter und KI sprechen könnten [10, 11]. So warnt z. B. Bryson [10], dass eine unbedachte Abtretung von Verantwortung an Roboter zu einer Vermenschlichung solcher Systeme und einer Entmenschlichung realer Personen führen könnte. Auf gesellschaftlicher Ebene könnte dadurch unternehmerische Verantwortung unterminiert und es könnten begrenzte Ressourcen an Roboter verschwendet werden, die ansonsten direkt Menschen und menschlicher Interaktion zugutekommen würden [10]. Zudem wird fehlende Gerechtigkeit im Zugang zu robotischen Systemen für ökonomisch benachteiligte Personen als Problem gesehen [12].

### 3. Das Ende der Privatsphäre.

Mit jeder digitalen Handlung produzieren wir neue Daten, die von Unternehmen, Regierungen und anderen Akteuren für Überwachungs- und Kontrollzwecke ausgenutzt werden können [2]. Darüber hinaus haben wir so gut wie kein Verständnis für die potenziellen Auswirkungen der Überwachung und Verfolgung sensibler Daten, wie beispielsweise Gesichtserkennungsdaten, Biometrie, genomische Daten und deren Bearbeitung mittels KI-gestützter prädiktiver Analysen [9].

Dieses grundsätzliche Szenario ist im Gesundheitsbereich besonders kritisch zu sehen, beispielsweise wenn Roboter und kognitive Systeme mit vulnerablen Gruppen wie Kindern oder in der Altenpflege zusammenarbeiten sollen. In jüngeren Arbeiten wurden dabei vor allem die Gefahren des Einsatzes von Begleitrobotern in der Altenpflege beschrieben [5, 11, 13]. Zu den führenden Autoren in dieser Debatte gehören Sharkey & Sharkey [14], die argumentieren, dass Roboter in der Altenpflege zu (1) einer Verringerung des menschlichen Kontakts, (2) einer Zunahme des Gefühls der Objektivierung und des Kontrollverlusts, (3) einem Verlust der Privatsphäre, (4) einem Verlust der persönlichen Freiheit, (5) einer Täuschung und Infantilisierung älterer Menschen und (6) einer Verringerung der Autonomie älterer Menschen führen werden. Bislang scheinen diese begründeten Bedenken in Nutzerstudien jedoch gegenüber eher ökonomisch motivierten Sorgen zurückzustehen, dass einkommensschwächere Nutzer:innen aus Kostengründen nicht gleichwertig von solchen Systemen profitieren könnten [12].

### 4. KI als Magnet menschlicher Aufmerksamkeit.

Die menschliche Fähigkeit, Belohnungen aufzuschieben, steht schon seit längerer Zeit unter dem Eindruck immer größer werdender digitaler und medialer Versuchungen [2]. Der sogenannte Marshmallow-Test [15] wird dabei oft als ein anschauliches Beispiel herangezogen, in dem es um die Bedeutung der Fähigkeit geht, süßen und kurzfristigen Versuchungen zunächst zu widerstehen (z. B. Marshmallows), um später eine größere Belohnung zu erhalten. Wie neuere Replikationen zeigen, mögen die unmittelbaren Auswirkungen der reduzierten Fähigkeit zum Belohnungsaufschub auf schulische Leistungen und spätere Verhaltensauffälligkeiten weniger ausgeprägt sein, als in den ursprünglichen Studien angenommen [16]. Dennoch leben wir bereits heute in einer tiefgreifend mediatisierten Gesellschaft, in der Medientechnologien weitgehend mit den alltäglichen Praktiken unserer sozialen Welt verflochten sind [17, 18]. Dadurch bekommen Plattformen, die durch entsprechende Algorithmen auf eine möglichst lange Verweildauer und Interaktionsintensität ausgerichtet sind, eine immer größere Anziehungskraft. Das Worst-Case-Szenario besteht dabei darin, dass wir durch diese automatisierten Mechanismen (z. B. Likes, Kommentare, Follower) immer mehr Zeit mit derartigen Plattformen verbringen und dadurch immer weniger Zeit für das Streben nach einem positiven, produktiven und erfüllten Leben haben [19].

Auch dieses Szenario und die damit verbundenen Herausforderungen sind im Gesundheitsbereich besonders bedeutsam. So können z. B. ältere Menschen mit Demenz nicht mehr immer vollumfänglich einschätzen, wann auf sie zugeschnittene Algorithmen sie von einer positiven und produktiveren Gestaltung ihres Alltags abhalten. Auf der anderen Seite kann es jedoch ethisch ebenso problematisch sein, nicht (mehr) neurotypische Erwachsene von der Nutzung neuer Technologien auszuschließen. Dies diskutieren beispielsweise Earp and Grunt-Mejer [20] anhand der Problematik der Nutzung von Sexrobotern im Alter. Einerseits könnten gerade ältere MmD von neuen Innovationen im Gesundheitsbereich besonders profitieren. Andererseits handelt es sich hier jedoch oft um besonders vulnerable Gruppen. Vorliegende ethische Konzepte sind hier immer noch primär auf den Fall von uneingeschränkt einwilligungsfähigen neurotypischen Erwachsenen ausgelegt [20].

### 5. KI als Treiber von Ungleichheit und Vorurteilen.

KI-gestützte Systeme übernehmen immer größere Teile unseres Alltagslebens [2]. Gleichzeitig zeigen diese Systeme jedoch weiterhin Schwächen, wenn es um die unvoreingenommene Berücksichtigung unterschiedlicher Erfahrungen und Merkmale verschiedener Menschen geht. So leiden Ansätze, die auf maschinellen Lernverfahren basieren, oft unter sogenannten „Biases“ (deutsch: Voreingenommenheiten), die mit gravierenden Nachteilen für die Betroffenen verbunden sein können [21]. Dies schlägt sich beispielweise in den Algorithmen sozialer Netzwerke wie Twitter nieder, wo z. B. durch Biases der Algorithmen in der Analyse von Nutzerprofilen soziale Vorurteile gegenüber Geschlechterstereotypen verstärkt werden können [21]. Diese algorithmischen Voreingenommenheiten gehen oft auf implizite Vorurteile der menschlichen Programmierer:innen zurück und lassen sich daher nur schwer vermeiden [2]. Ebenso können entsprechende Verzerrungen durch unzureichende Datengrundlagen z. B. während des Trainings der Modelle entstehen, da hier z. B. Minderheiten oft nur unzureichend vertreten sind. In diesem Worst-Case-Szenario könnten somit insbesondere Minderheiten in Zukunft noch stärker marginalisiert und ausgegrenzt werden. Da KI Probleme auf der Grundlage „vortrainierter“ Modelle und Daten und nicht auf der Basis der einzigartigen Bedürfnisse jedes Einzelnen löst, erzeugen solche Systeme ein Maß an Konformität, das es in der menschlichen Gesellschaft nicht gibt. KI könnte sich so in eine Art „kafkaesken Torwächter“ [2] verwandeln, der den Zugang zu Kundendienst, Arbeitsplätzen, Gesundheitsversorgung und vielem mehr verwehrt.

Während sich die möglichen Auswirkungen dieses Szenarios auf die gesamte Breite des sozialen Lebens erstrecken, sind auch hier die möglichen Nachteile im Gesundheitsbereich besonders kritisch zu sehen. Im Falle einer „Algokratie“ [22], in der Algorithmen zunehmend wichtige öffentliche Entscheidungen beeinflussen oder gar selbst treffen, könnten z. B. bereits anderweitig besonders benachteiligte Gruppen zunehmend von grundlegenden Gesundheitsdienstleistungen ausgeschlossen werden.

### 6. Die Angst vor Künstlicher Intelligenz.

Da KI zunehmend mächtiger wird, ist auch mit Blick auf die bereits genannten Szenarien mit weiter zunehmenden Ängsten und auch mit mehr Kontrolle, z. B. seitens der Regierungen zu rechnen [2]. Während eine konsequente Weiterentwicklung der technischen, rechtlichen und ethischen Rahmenbedingungen von KI somit dringend geboten ist, gibt es auch hier eine Kehrseite von übereilten und unüberlegten Verboten: Allein aus Angst vor KI könnten der Menschheit alle oder viele ihrer Vorteile entgehen. Der notwendige Schutz vor unbeabsichtigten negativen Folgen von KI könnte so dazu führen, dass wir uns weigern, KI für das tatsächlich Gute zu nutzen, das sie bewirken kann [2].

Auch dieses Szenario gilt insbesondere für Anwendungen im Gesundheitsbereich, wo beim Verzicht auf den Einsatz von KI, z. B. aus Datenschutzgründen oder zum Schutz der Privatsphäre, auch nachteilige langfristige Auswirkungen fehlender KI-basierter Interventionsmöglichkeiten unbedingt mitbedacht werden sollten.

## Vom Worst Case zum Best Case der KI-Nutzung im Gesundheitsbereich

Bestimmte Risiken des Einsatzes von KI wie z. B. Täuschung und der Verlust von Autonomie im Alltag werden einhellig als gravierende Probleme gesehen [10, 23]. Viele der bereits angesprochenen ethischen Herausforderungen zeigen sich dabei im Gesundheitsbereich in besonderer Schärfe. In der Folge könnte bei bestimmten Fragestellungen ein Umdenken erforderlich werden. Wie müssten kognitive Systeme gestaltet werden, um Menschen in ihrem Alltag zu unterstützen, ohne ihre Autonomie zu untergraben? Anknüpfend an unsere bisherige Risikoanalyse stellen wir in diesem Abschnitt die Frage nach dem „Best Case“, in dem KI die Handlungsspielräume und Autonomie aktiv und positiv erweitert.

Zahlreiche moderne ethische Überlegungen basieren noch immer auf der Vorstellung einer neurotypischen „normalen“ erwachsenen Person, deren Autonomie es zu schützen gilt [20]. So besagen unsere vorherrschenden ethischen Modelle beispielsweise, dass ethisch richtiger Sex weitgehend durch die gegenseitige Zustimmung zwischen Erwachsenen definiert wird, während eine fehlende Zustimmung Sex als falsch erscheinen lässt [24]. Was geschieht jedoch, wenn die Autonomie eines Menschen absehbar durch Faktoren wie etwa Demenz eingeschränkt wird? Dies kann bedeuten, dass dieser ab einem bestimmten zukünftigen Zeitpunkt nicht mehr ohne weiteres seine Zustimmung geben kann. Manche Entscheidungen oder Hilfestellungen könnten hier jedoch unter bestimmten Bedingungen an intelligente adaptive Systeme delegiert werden, um hilfsbedürftigen Menschen in der Summe zu mehr Autonomie zu verhelfen. Hier schlägt beispielsweise Bianchi [25] vor, ältere Menschen mit Demenz im Vorfeld zu fragen, ob und/oder wann sie einen Sexroboter in der Zukunft benutzen würden, falls eine solche Möglichkeit verfügbar werden sollte. Wie durch dieses vielleicht immer noch etwas ungewöhnliche Beispiel illustriert wird, besteht bei einem ethisch gut durchdachten Einsatz von KI-gestützten Systemen somit durchaus die Möglichkeit, die Autonomie und Lebensqualität benachteiligter Menschen nachhaltig zu stärken. Womöglich wird dies jedoch eine langfristige Sicht über die gesamte Lebensspanne der Nutzer:innen erfordern. In unserer Vision eines Best-Case-Szenarios würde KI somit menschliche Autonomie über lange Zeiträume und Lebensphasen hinweg betrachten, prädizieren und unterstützen können.

Tatsächlich lässt sich dieser Ansatz aus unserer Sicht zukünftig auf zahlreiche Anwendungsbereiche im Gesundheitswesen übertragen. So könnte KI gezielt dazu eingesetzt werden, die autonomen Entscheidungen, Werte und Bedürfnisse bzgl. Privatsphäre von Menschen über einen langen Zeitraum hinweg zu erlernen und ggf. auch an andere Akteur:innen zurückspiegeln. Ein mögliches Einsatzfeld läge hier beispielsweise im Bereich der häuslichen Pflege, in der KI-basierte Systeme Angehörige von demenzerkrankten Personen entlasten könnten, indem sie wechselndes Pflegepersonal über die erlernten Präferenzen und Autonomiebedürfnisse der pflegebedürftigen Person informieren und damit der Überforderung vorbeugen. Ein auf langjährige Nutzung hin ausgerichtetes Assistenzsystem könnte einen erheblichen Beitrag zu einer besser auf die jeweilige Person zugeschnittenen Pflege leisten. In unserer Vision eines Best-Case-Szenarios des ethischen Einsatzes von KI im Gesundheitswesen ließen sich das Bedürfnis nach Hilfe und jenes nach Autonomie weitgehend vereinbaren.

Die Abwägung zwischen Hilfe und Autonomie stellt sich auch für die helfenden Personen: Was wünscht sich der oder die Hilfebedürftige und welche konkreten Bedürfnisse sind gerade besonders wichtig? Hier könnten insbesondere biosignalbasierte adaptive Systeme [26] einen Beitrag leisten, indem sie z. B. helfen, die verbleibenden sprachlichen und kommunikativen Fähigkeiten einer erkrankten Person zu unterstützen [27–30]. Ebenso könnten diese Systeme lernen zu erkennen, durch welche Angebote und Anregungen die an Demenz erkrankte Person wieder zu mehr aktiver Teilnahme (englisch: „Engagement“) am Alltagsgeschehen angeregt werden könnte [31–34].

Statt des Szenarios eines fortwährenden Spagats zwischen Ethik und KI im Gesundheitswesen sehen wir somit ein erhebliches Potenzial einer sich über die gesamte Lebensspanne hinweg entwickelnden Symbiose, die dem Erhalt der menschlichen Autonomie dient. Allen dystopischen Visionen in diesem Feld zum Trotz könnten KI und Roboter in Zukunft sehr viel mehr als Freunde denn als Feinde der Menschheit agieren [13, 35, 36]. Diese Perspektive nehmen auch große aktuelle Initiativen ein, die entsprechende Worst-Case-Szenarien verhindern helfen sollen. Ein Beispiel ist die interdisziplinär aus Vordenkern aus der Wissenschaft, Industrie, Zivilgesellschaft und Politik zusammengesetzte Initiative „Ethical Aligned Design“ (EAD) [37]. In dieser Initiative haben mehrere hundert Fachleute aus technischen und geisteswissenschaftlichen Disziplinen aus sechs Kontinenten gesellschaftliche und politische Leitlinien formuliert, damit autonome und intelligente Systeme menschenzentriert bleiben und den Werten und ethischen Prinzipien der Menschheit dienen.

Die Prinzipien des EAD verfolgen das Ziel der Weiterentwicklung von KI bei ausdrücklicher Achtung unserer unveräußerlichen Grundrechte und unserer Würde sowie die Steigerung des menschlichen Wohlergehens und der ökologischen Nachhaltigkeit. EAD definiert zu diesem Zweck zunächst acht allgemeine Grundsätze, die von den Entwickler:innen von KI-Systemen zu befolgen sind: Menschenrechte, Wohlbefinden, Datenautorität, Effektivität, Transparenz, Verantwortlichkeit, Bewusstsein und Kompetenz. Darüber hinaus bietet es klare Richtlinien, Methoden und Messgrößen, mit denen diese allgemeinen Prinzipien in die Praxis umgesetzt werden können [37].

Seit 2015 wird an der Universität Bremen ein KI-System zur Unterstützung von MmD entwickelt, welches ursprünglich im Rahmen des „I-CARE“ Projekts (2015–2018) entstanden ist. Bei der Konzeption, Umsetzung und Nutzung des „I-CARE-Systems“ [32, 38–40] haben sich die beteiligten Teams an die Grundsätze und Leitlinien des EAD gehalten. Auch in den weiterführenden Forschungen arbeiten die Teams konsequent daran, die Student:innen für die ethischen Überlegungen im Zusammenhang mit KI-Systemen im Allgemeinen und mit adaptiven Assistenzsystemen im Besonderen zu sensibilisieren, indem diese Themen in der Lehre und in der Ausbildung besprochen werden. Im Folgenden wird die Entwicklung des I‑CARE Systems beispielhaft beschrieben, um unsere Vision der Entwicklung hin zu einem möglichen Best Case möglichst anschaulich zu illustrieren.

## I-CARE: ein KI-System für Menschen mit Demenz

Demenz beschreibt eine breite Gruppe krankheitsbedingter Symptome, die sich auf Gedächtnis, Verhalten, Denken und soziale Fähigkeiten auswirken [41]. Diese Symptome beeinträchtigen die Aktivitäten des täglichen Lebens und die soziale Autonomie von MmD erheblich und wirken sich zudem negativ auf die Gesundheit und das Wohlbefinden der sie pflegenden Menschen aus. Beispielsweise sind diese oft selbst von Depressionen betroffen [42]. Es besteht somit ein dringender Bedarf an Gesundheitsförderung sowie primärer und sekundärer Prävention für diese Bevölkerungsgruppe. Da jedoch bis heute keine Heilungsmöglichkeiten für Demenz bekannt sind, darf die Prävention auf tertiärer Ebene nicht bagatellisiert werden [32, 43]. Die tertiäre Prävention bei Demenz zielt darauf ab, die Lebensqualität von MmD und ihren Betreuer:innen positiv zu beeinflussen, indem die negativen Auswirkungen der Krankheitssymptome gemildert werden. Der Einsatz von KI in der Pflege von Demenzerkrankten könnte die globale Belastung durch Demenz verringern und neuartige Technologien ermöglichen, die die Lebensqualität von MmD und ihren Pfleger:innen verbessern [39]. Das Tablet-basierte Aktivierungssystem I‑CARE ist ein aktuelles Beispiel für eine Maßnahme zur tertiären Prävention.

I‑CARE ist ein Empfehlungssystem, das verschiedene Arten von Stimuli bietet, die auf die Aufrechterhaltung oder Verbesserung der allgemeinen kognitiven und sozialen Funktionen von MmD abzielen [39]. Das System ist dabei gezielt so konzipiert, dass es gemeinsam von MmD und ihren formellen und informellen Betreuer:innen genutzt werden kann. I‑CARE bietet einen Pool von 346 Aktivierungsinhalten unterschiedlicher Art, z. B. Bildergalerien, Videos, Audios, Quiz, Spiele, Phrasen und Texte. Damit bietet das System Aktivitäten, die unterschiedliche Ebenen der kognitiven Verarbeitung ansprechen. Die einzelnen Aktivierungsinhalte beziehen sich dabei auf verschiedene Themen des täglichen Lebens, die speziell für diese Benutzergruppe ausgewählt wurden (z. B. Gartenarbeit, Sport oder Backen).

**Figure 1:**
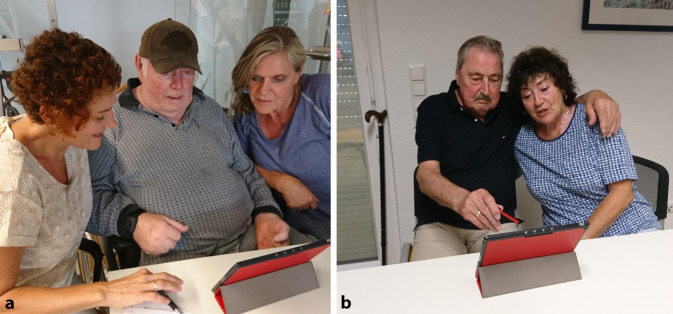

Zur individualisierten Auswahl geeigneter Aktivierungsinhalte schätzt I‑CARE fortlaufend Aktivierungszustände und das Engagement der Nutzer:innen mittels maschineller Lernverfahren ein. Diese Analyse erfolgt anhand multimodaler Daten. Mithilfe der Kamera und des Tabletmikrofons erfasst das System Video- und Audiosignale. Die grafische Benutzeroberfläche registriert die Interaktionsereignisse und ein E4-Armband^1^ dient zur Erfassung physiologischer Reaktionen. Nach jedem Aktivierungsinhalt bittet das System die MmD um eine Bewertung, wie gut ihnen die Aktivierung gefallen hat („Hat Ihnen der Inhalt gefallen?“) auf einer Smiley-Bewertungsskala (positiv, neutral, negativ). Abb. 1 zeigt zwei beispielhafte Situationen aus den Aktivierungssitzungen.

Die Empfehlungen des I‑CARE-Systems werden individualisiert auf Grundlage der Ähnlichkeiten eines gegebenen Benutzerprofils mit allen möglichen Aktivierungsinhalten erstellt. Die Ähnlichkeit basiert auf dem minimalen Abstand zwischen den zugehörigen Kennzeichen (englisch: „tags“) innerhalb des semantischen Netzwerkes *ConceptNet* [34, 44]. Es wird eine Empfehlungsliste erstellt, deren Aktivierungsinhalte in absteigender Reihenfolge nach den Ähnlichkeiten des Benutzerprofils mit jedem Inhalt des Netzwerks sortiert sind. Negativ bewertete Aktivierungsinhalte werden ans Ende der Liste verschoben, während Inhalte, die bereits gesehen wurden, aus der Liste entfernt werden. Für jede neue Bewertung wird der Bewertungsdurchschnitt des Benutzerprofils neu berechnet und die Bewertungen werden erneut in positive und negative aufgeteilt [39]. Wenn sich die Menge der Tags in einem Benutzerprofil ändert, werden die Ähnlichkeiten zwischen dem Benutzerprofil und den Aktivierungsinhalten ebenfalls neu berechnet. Es hat sich dabei als sehr erfolgreich erwiesen, die Teilnehmer:innen mit Aktivierungsinhalten zu versorgen, die auf ihren früheren Vorlieben basieren [32].

Im Laufe der Nutzung lernt das Empfehlungssystem von I‑CARE so, die individuellen Bedürfnisse und Ressourcen seiner Nutzer:innen besser einzuschätzen, und personalisiert automatisch die Aktivierungsinhalte [39]. Darüber hinaus können Betreuer:innen vor Ort über die I‑CARE-Fernanruffunktion mit Fachleuten in Verbindung treten, um Aktivierungssitzungen in Echtzeit zu unterstützen. Auf diese Weise bietet I‑CARE die technische Grundlage für eine dezentralisierte und spontane Bildung von Ad-hoc-Aktivierungsgruppen und fördert eine enge Einbeziehung des sozialen Netzwerks und der Betreuungsgemeinschaft. I‑CARE fördert so neue Pflegeinfrastrukturen in der Gemeinde und in der Nachbarschaft und entlastet professionelle und informelle Pflegekräfte [39].

## Ethische Erwägungen für das KI-System I-CARE

Um den in den Worst-Case-Szenarien dargestellten Problemen zu begegnen, wurde bei der Entwicklung des I‑CARE-Systems konsequent daran gearbeitet, eine ethisch verantwortungsvolle Umsetzung des Systems im Sinne der spezifischen Anforderungen der Pflege von Demenzerkrankten [45] zu gewährleisten. Als Teil der Bereitstellung eines automatischen Empfehlungssystems erfasst das I‑CARE-System biografische Informationen sowie Biosignale. Informationen wie Alter, (früherer) Beruf und Interessen werden im individuellen Profil der Benutzer:innen gespeichert [39]. Zudem werden Biosignale wie Sprache, Mimik oder physiologische Signale erfasst, die Aufschluss über emotionale Reaktionen und den Grad der Beteiligung eines MmD an den durch I‑CARE vorgeschlagenen Aktivierungen geben können [40]. Aufgrund des privaten Charakters dieser Art von Daten folgt I‑CARE neben den üblichen Anforderungen an ein ethisch verantwortliches Studiendesign (z. B. Befürwortung durch eine Ethikkommission) den folgenden grundlegenden ethischen Erwägungen für KI im Gesundheitswesen.

### Transparenz.

In I‑CARE bezieht sich die Transparenz auf die Verfügbarkeit von verständlichen Informationen für jede Person, die das I‑CARE-System implementiert oder nutzt, einschließlich Versuchsleiter:innen, Pflegekräften in unterstützenden Rollen oder MmD als Nutzer:innen. Alle an der Umsetzung von I‑CARE beteiligten Akteur:innen haben Zugang zu Informationen über die Art und den Zweck aller gesammelten Daten sowie darüber, wie diese Daten genutzt werden, um empfohlene Aktivierungsinhalte zu erstellen [33, 39]. Teilnehmerinformationen zu komplexen Themen erfordern besondere Überlegungen, wenn sie an Menschen mit kognitiven Beeinträchtigungen angepasst werden sollen. Bei der Entwicklung und Evaluierung von I‑CARE wurden diese Informationen an einen Demenzkontext angepasst, da auch Menschen mit Demenz ein Recht auf Informationen über Dinge haben, die sie betreffen [46]. Die Darbietung erfolgt in einer möglichst leicht verständlichen Form. Die Ziele und Funktionen des I‑CARE-Systems und die Aufzeichnung der (Biosignal‑)Daten werden so weit wie möglich entsprechend den Fähigkeiten der MmD und ihrer Betreuer:innen erklärt [39]. Dazu gehören Anpassungen der Sprache, des Stils, der Länge und des Formats der Informationen, die im I‑CARE-System und in den Zusatzmaterialien enthalten sind. Schließlich werden alle Informationen auch mündlich in Nachbesprechungen an die Betroffenen und ihre Betreuer weitergegeben.

### Inklusivität.

Um dem Risiko systematischer Verzerrungen bei der automatischen Erkennung von „Engagement“ zu begegnen, werden Algorithmen benötigt, die das Engagement der Teilnehmer:innen unabhängig von Merkmalen wie z. B. Alter und Hautfarbe erkennen können. Inklusivität erfordert dabei insbesondere, dass die einzusetzenden Modelle bereits zum Zeitpunkt des Trainings der Algorithmen mit entsprechenden Daten „gefüttert“ werden. Der Datensatz, der für das Training des I‑CARE-Systems zur Erkennung von Engagement verwendet wurde, bestand aus multimodalen Daten älterer Menschen mit Demenz, inklusive Video, Audio- und Biosignaldaten wie der über das E4-Armband erfassten Hautleitfähigkeit. Zum jetzigen Zeitpunkt [32] muss das System jedoch als voreingenommen betrachtet werden, da der Trainingsdatensatz nur aus deutschsprachigen Teilnehmer:innen mit heller Hautfarbe besteht. Es wird daher ein noch größerer und diverserer Datensatz benötigt, der Menschen verschiedener Ethnien und Nationalitäten umfasst, um diesem wichtigen Grundsatz vollends gerecht zu werden.

### Gleichberechtigung.

Unsere Überlegungen zur Gleichberechtigung beim Einsatz von KI-Technologie wie I‑CARE in der Pflege von Demenzerkrankten zielen darauf ab, Personen mit unterschiedlichen Voraussetzungen eine gleichberechtigte Nutzung der Technologie zu ermöglichen. Dazu gehört das Bestreben, den Zugang und die konsequente Nutzung für alle sozioökonomischen Schichten zu maximieren [45]. Obwohl dieses Ziel aufgrund der Neuartigkeit von KI-Anwendungen im Bereich der öffentlichen Gesundheit nicht kurzfristig erreicht werden kann, sollten KI-Systeme wie I‑CARE für alle gängigen digitalen Gerätetypen und Dienste verfügbar gemacht werden.

### Menschenrechte, Autonomie, Datenhoheit.

Die aufgezeichneten Biosignale und biografischen Daten werden in pseudonymisierter Form verarbeitet und archiviert. Die persönliche Autonomie wird maximiert, wenn die Endnutzer:innen keine passiven Objekte eines Top-down-Designs sind, sondern die KI-Technologie an ihre Bedürfnisse angepasst wird [45]. Dafür wurde I‑CARE in enger Zusammenarbeit mit MmD, formellen und informellen Betreuer:innen entwickelt [39]. Darüber hinaus können Einzelpersonen ihr Benutzerprofil spezifizieren, indem sie persönliche und biografische Informationen angeben, z. B. ihren früheren Beruf. Auf dieser personalisierten Grundlage kann dann das I‑CARE-System geeignete Aktivierungsinhalte empfehlen. Diese Informationen werden in einem hochsicheren IT-System gespeichert, um die Privatsphäre und Vertraulichkeit des Einzelnen bestmöglich zu schützen.

### Verantwortung und Rechenschaftspflicht.

Für das I‑CARE-System liegen Verantwortung und Rechenschaftspflicht eindeutig bei dem Forschungsteam, welches das System entwickelt und umgesetzt hat. Daher wurde frühzeitig ein strenger Prozess zur Entwicklung ethischer Richtlinien und Leitprinzipien durchgeführt, an denen sich alle Akteur:innen des interdisziplinären Konsortiums orientieren konnten. Das Konsortium umfasste Geriater:innen, Gerontolog:innen, Psycholog:innen, Gesundheitswissenschaftler:innen und Krankenpfleger:innen sowie Ingenieur:innen und Informatiker:innen [39]. Durch die Bereitstellung ansprechender Aktivierungsinhalte dienen Systeme wie I‑CARE aus unserer Sicht beispielhaft dem in diesem Beitrag diskutierten Ziel des Erhalts und der Unterstützung von persönlicher Autonomie sowie dem Erfolg der tertiären Prävention von Krankheiten wie Demenz.

## Fazit

Der Einsatz von KI führt insbesondere dann zu einem Spagat, wenn ethische Aspekte nicht bereits im Vorfeld nachvollziehbar abgewogen werden. Unsere vorläufige Analyse möglicher Worst-Case-Szenarien verdeutlicht dabei unter anderem Gefahren durch unkontrollierte oder übergriffige KI, Desinformationen, fehlende Datensicherheit und Biases im Bereich des Trainings maschineller Lernverfahren. Insbesondere im Gesundheitsbereich ergibt sich die zentrale Herausforderung, menschliche Autonomie zu fördern und über die Lebensspanne zu erhalten. Ein „blinder“ Einsatz von KI birgt hier besondere Gefahren, weil ihre Unterstützung ab einem bestimmten Punkt zu leichtfertig eingesetzt werden könnte, da KI im Vergleich zu endlicher menschlicher Arbeitskraft nur sehr wenige Ressourcen benötigt. Ähnlich wie z. B. bei einem Rollstuhl kann hier stets die Versuchung bestehen, das Hilfsmittel auch dann einzusetzen, wenn es gar nicht (mehr) benötigt wird. Andererseits ist der Nutzen bei bedarfsgerechtem Einsatz solcher Hilfsmittel offensichtlich. KI sollte ihre Nutzer:innen deshalb möglichst individualisiert und situationsgerecht unterstützen, um Autonomie zu fördern, anstatt sie zu gefährden. Die große Herausforderung für die Entwicklung ethischer KI im Gesundheitsbereich ist somit die Entwicklung von Intelligenz, die sich auch bedarfsgerecht selbst zurücknehmen kann. Dies sollte ethisch abgewogen und im Einverständnis mit den jeweiligen Nutzer:innen und möglichst vorausschauend geschehen. Mit Blick auf Beispiele wie das hier kurz skizzierte I‑CARE-Projekt sind wir insgesamt zuversichtlich, dass die nächste Generation von KI im Gesundheitswesen diesen Herausforderungen sehr viel besser gerecht werden könnte.

## Footnotes

1 https://www.empatica.com/.
